# The state of the art in the treatment of severe aplastic anemia: immunotherapy and hematopoietic cell transplantation in children and adults

**DOI:** 10.3389/fimmu.2024.1378432

**Published:** 2024-04-05

**Authors:** Agnieszka Piekarska, Katarzyna Pawelec, Anna Szmigielska-Kapłon, Marek Ussowicz

**Affiliations:** ^1^ Department of Hematology and Transplantology, Medical University of Gdansk, Gdansk, Poland; ^2^ Department of Oncology, Pediatric Hematology, Clinical Transplantology and Pediatrics, Medical University of Warsaw, Warsaw, Poland; ^3^ Department of Hematology, Medical University of Lodz, Lodz, Poland; ^4^ Department of Pediatric Bone Marrow Transplantation, Oncology and Hematology, Wroclaw Medical University, Wroclaw, Poland

**Keywords:** aplastic anemia, antithymocyte globulin, eltrombopag, hATG, hematopoietic cell transplantation, immunosuppression, rATG

## Abstract

Acquired aplastic anemia (AA) is an immune-mediated bone marrow (BM) failure where marrow disruption is driven by a cytotoxic T-cell–mediated autoimmune attack against hematopoietic stem cells. The key diagnostic challenge in children, but also in adults, is to exclude the possible underlying congenital condition and myelodysplasia. The choice of treatment options, either allogeneic hematopoietic cell transplantation (alloHCT) or immunosuppressive therapy (IST), depends on the patient’s age, comorbidities, and access to a suitable donor and effective therapeutic agents. Since 2022, horse antithymocyte globulin (hATG) has been available again in Europe and is recommended for IST as a more effective option than rabbit ATG. Therefore, an update on immunosuppressive strategies is warranted. Despite an improved response to the new immunosuppression protocols with hATG and eltrombopag, some patients are not cured or remain at risk of aplasia relapse or clonal evolution and require postponed alloHCT. The transplantation field has evolved, becoming safer and more accessible. Upfront alloHCT from unrelated donors is becoming a tempting option. With the use of posttransplant cyclophosphamide, haploidentical HCT offers promising outcomes also in AA. In this paper, we present the state of the art in the management of severe AA for pediatric and adult patients based on the available guidelines and recently published studies.

## 1 Introduction

Bone marrow failure (BMF) syndromes are a consequence of inherited or acquired conditions, resulting in hematopoiesis failure. The hematopoietic stem cell (HSC) compartment in BMF is disrupted either by constitutional mutations of genes involved in hematopoiesis, or through a direct destruction of HSCs by cytotoxic agents or an immune-mediated attack.

Aplastic anemia (AA) is a hematological entity associated with bone marrow (BM) aplasia and hematopoiesis failure, which was first described at the turn of the 19th and 20th centuries (1). The historical term is slightly confusing, as most patients experience pancytopenia in addition to anemia, while “aplastic” defines the inability of the BM to effectively produce blood cells as a result of various mechanisms. The key diagnostic challenge is to identify the underlying condition involved in aplasia in individual patients to choose the optimal treatment option: either allogeneic hematopoietic cell transplantation (alloHCT) or immunosuppressive therapy (IST).

The diagnostic hallmark of AA is the presence of at least two-lineage cytopenia with hemoglobin levels lower than 10 g/dL, platelet count lower than 50 × 10^9^/L, or neutrophil count lower than 1.5 × 10^9^/L (2). The modified Camitta criteria classified AA into three categories on the basis of BM cellularity, an absolute neutrophil count (ANC), platelet count, and reticulocyte count (3). Severe AA (SAA) is characterized by pancytopenia with ANC lower than 0.5 × 10^9^/L, platelet count lower than 20 × 10^9^/L, reticulocyte count lower than 20 × 10^9^/L (manual count) or 60 × 10^9^/L (using an automated analyzer), and less than 25% of normal cellularity in BM biopsy. Very severe AA (vSAA) is defined by the same criteria except ANC of less than 0.2 × 10^9^/L. Patients not fulfilling the SAA/vSAA criteria with BM cellularity are classified as having nonsevere AA, but many patients in this category have other BMF syndromes that require a diagnosis.

## 2 Pathophysiology and clonal evolution

In short, AA is an acquired immune-mediated BMF syndrome where marrow disruption is driven by a cytotoxic T-cell–mediated autoimmune attack against HSCs sustained by type I interferons that polarize the immune system toward T-helper 1 responses in early phases and then toward T-helper 17 and effector memory CD8+ T-cell in late stage and severe disease (4). The immune-mediated factors play a central role in the pathogenesis of hepatitis-associated AA – a distinct variant of AA with a prevalence of about 5%, in which pancytopenia appears 2 to 3 months after acute hepatitis and shows quite good response to IST of 55% to 70% in patients who lack an optimal HSC donor (5–8).

Among multiple studied factors, the dysfunction of BM environment and mesenchymal stromal cell dysregulation might contribute to the damage in the stem-cell niche (9). Immune pressure from T-cell–mediated mechanisms leads to somatic gene alterations in HSCs, resulting in immune evasion (10). The telomere length in AA patients was reported to be significantly shorter than in age-matched controls (11, 12). Approximately 35% of patients with AA show telomere length shortening in granulocytes and mononuclear cells, which results in HSC exhaustion and is associated with the risk of relapse, clonal evolution, and worse overall survival (OS) (13, 14). In turn, telomere shortening due to mitotic demand in the HSC compartment can destabilize the genome by chromosome rearrangement and aneuploidy, which negatively affects therapy response and survival (14–16).

Fattizzo et al. reported a high prevalence of paroxysmal nocturnal hemoglobinuria (PNH) clones caused by somatic *PIGA* mutations in patients with AA and other hematological disorders (17). Next-generation sequencing (NGS) allowed the identification of somatic mutations responsible for clonality in AA even in small subclones. Moreover, different mutations in the same *PIGA* gene in a single patient supports the hypothesis that the mutations are acquired independently in different clones, promoting their expansion and evolution. The carriers of somatic *PIGA* and *BCOR/BCORL1* mutations show a lower risk of AA transformation to myelodysplastic syndrome (MDS), whereas myeloid driver lesions such as *DNMT3A* and *ASXL1* mutations characterize the group with a higher risk of malignancy (18). While most patients with AA have normal BM karyotype, trisomy 8 and del(13q) were reported and were linked to a more favorable response to therapy (19, 20). Uniparental disomy (UPD) of chromosome 6p (a locus containing the human leukocyte [HLA] cluster) can be identified in up to 11% of AA patients and is highly specific for AA, but not for other BMF syndromes (21, 22). In contrast, the presence of monosomy 7 in BMF/SAA suggests the diagnosis of MDS and a higher probability of constitutional disorders, such as *GATA2* deficiency, *SAMD9/SAMD9L*-related disorders, or telomer biology disorders (23, 24). In adults with AA, cytogenetic abnormalities at diagnosis are observed in 12% to 15% of patients, with the following findings reported in the literature: trisomies (+8, +6, +15), aberrations of chromosomes 7 and 13, UPD(6p), and del(5q) (25, 26).

Poor-risk mutation carriers with AA have a 40% higher risk of clonal evolution to MDS or acute myeloid leukemia (AML) compared with patients with *PIGA*, *BCOR*, and *BCORL1* mutations (27–30). Apart from the risk of clonal evolution, adverse effects in terms of response to IST, risk of relapse, and inferior OS were observed in patients without a PNH clone, with poor-risk mutations (*ASXL1*, *TP53*, *RUNX1*, *DNMT3A*), and shortened telomeres (31, 32). However, a detailed NGS-based analysis of the patients participating in the RACE study showed fluctuations in the variant allele frequency of detected mutations, and no mutation correlated with response to IST or OS (33).

## 3 Diagnosis

The diagnosis of AA must include the differentiation between the various pathologies resulting in pancytopenia and BMF syndromes. In histopathological BM assessment, hypoplastic MDS, acute leukemia, T-cell large granular lymphocyte leukemia, cancer metastases, and reticulin fibrosis must be excluded and BM cellularity should be clearly described. Diagnostic BM workup includes cytogenetic examination with fluorescent *in situ* hybridization (FISH) (1, 30, 34, 35). Immunophenotyping from peripheral blood (PB) directed to detect PNH is obligatory as PNH clone is observed in almost half of the patients with AA and has clinical implications (31, 36, 37). Evaluation in the direction of hepatitis-derived marrow failure including classic hepatitis viruses but also parvovirus B19, CMV, and EBV is also recommended at diagnosis (7).

In the differential diagnosis of AA, other conditions leading to BMF must be considered. Direct BM damage can be caused by radiation, chemotherapy, or exposure to toxic compounds such as benzene, and various drugs including chloramphenicol, gold compounds, nonsteroid anti-inflammatory drugs, and anticonvulsants (38, 39).

There is a high prevalence of inherited BMF syndromes in children and young adults. Constitutional BMF can be a manifestation of germline defect resulting from a loss of function of one or more of the genes involved in DNA repair (Fanconi anemia), telomere maintenance (dyskeratosis congenita and other telomeropathies), and deficiencies leading to hematopoiesis regulation (*GATA2*, *CTLA4*) (1). Genomic screening for constitutional syndromes and telomere length measurement (if available) should be done, and the presence of somatic mutation should be evaluated (40).

Hypocellular MDS can mimic the presentation of AA and evolve from various BMF syndromes, and the differentiation between these entities can pose a practical challenge. In a recent study from the United States, including 732 children and adult HCT recipients clinically diagnosed with acquired AA, a comprehensive genetic analysis using NGS identified 48 patients (6.6%) with unrecognized inherited BMF syndromes who had 62 germline pathogenic variants in 22 genes associated with inherited BMF syndromes (41). Moreover, patients with unrecognized inherited BMF syndromes had worse survival because of treatment-related mortality from organ failure.

Adults are less likely than children to have constitutional BMF, but the possibility should not be neglected because incomplete gene penetrance and variable expressivity do not always lead to physical abnormalities and might manifest solely with BMF. However, usually a family history is suggestive. Considering better access to NGS, a panel including *ASXL1*, *DNMT3A*, *BCOR*, *BCORL1*, *PIGA*, *RUNX1*, *TP53* and *GATA2* genes can be done.

To sum up, the diagnostic workup recommendations for SAA were presented by the Severe Aplastic Anemia Working Party of the European Society for Blood and Marrow Transplantation (EBMT) in 2013 and the North American Pediatric Aplastic Anemia Consortium in 2021 (35, 42). Due to a wide range of diagnostic recommendations, only key points should be mentioned. In addition to standard laboratory testing, as well as bone marrow aspirate and biopsy, patients should be evaluated for bone marrow karyotype, FISH for abnormalities of chromosomes 7, 8, 5, and del20q, presence of the PNH clone, chromosomal breakage and/or genetic Fanconi anemia testing, and telomere length (with flow FISH techniques). The availability of NGS testing prompts early evaluation for germline mutations in the *GATA2*, *SAMD9/9L*, *ERCC6L2*, and *EVI1* genes, telomere biology disorders/dyskeratosis congenita, Fanconi anemia, ribosomopathies (Shwachman-Diamond syndrome, Diamond-Blackfan anemia) when appropriate, but especially in children and young adults or patients with atypical or nonhematological manifestations. Although not used in current clinical algorithms, the NGS panel to detect the presence of somatic variants (consisting typically of *PIGA*, *DNMT3A*, *ASXL2*, *TET2*, *BCOR*, *BCORL*, *NPM1*, *ETV6*, *TP53*, but not limited to this list) might be integrated in the nearest future but their clinical value has to be validated (43). The growing knowledge on monogenic defects resulting in one- or multilineage cytopenias and rapid progress in this field warrant clinical alertness and unhindered referral for genetic testing (44). Patients should undergo the work-up for autoimmune cytopenias with ANA and infectious screening.

## 4 History of AA therapy

Initially, the treatment of patients with AA consisted of supportive transfusions and the administration of steroids and vitamins. Historically, the first effective pharmacotherapy of AA was developed in the 1960s and was based on androgen administration (45). Thus, androgen therapy has established its place in the therapy of AA, but was associated with multiple adverse effects, such as virilization, jaundice and hepatotoxicity, hyperlipidemia, and behavioral effects with no improvement in mortality (46, 47). Although alloHCT and IST are highly effective in AA, androgens continue to be an option, even in the frontline, for transplant ineligible patients with no access to modern IST (46).

In 1970s, alloHCT was proved to be more effective than conventional therapies, thus becoming the cornerstone of the curative treatment of AA (48, 49). Currently, alloHCT is the widely accepted and recommended first-line therapy in children with matched sibling donors (MSDs), and fit young adults. The increasing availability of matched unrelated donors (MUDs) has led to the concept of upfront alloHCT from MUDs, but the time to arranging transplantation in this situation is longer and can reach 2 to 3 months.

In the early 1980s, another therapeutical option was offered for patients with AA. The phenomenon of alloHCT rejection with autologous reconstitution resulting in hematological remission of AA warranted studies of immunosuppression. The antilymphocyte globulin (ALG) was studied in different clinical settings (with or without alloHCT or androgens). For ALG alone, the treatment showed the response rates ranging from 30% to 40% (50, 51). Considering the immune origin of the disorder, intensive IST with antithymocyte globulin (ATG) was also evaluated (52, 53). Antithymocyte globulin contains polyclonal antibodies directed against different T-cell antigens including CD2, CD3, CD4, CD8, CD11, CD18, CD25, and others. Treatment with ATG results in the anergy of T-lymphocyte cells and induces their depletion via antibody-dependent cell-mediated cytotoxicity. Complement-dependent lysis is an additional mechanism resulting in T-cell lysis. The mechanisms of action of ATG were reviewed by Mohty et al. (54). Since 2022, horse ATG (hATG) has been available again in Europe and is recommended for IST. Therefore, the update of immunosuppressive strategies is warranted.

The next step towards improvement of the outcomes was made with implementation of eltrombopag (ELT), an oral thrombopoietin receptor agonist (TPO-RA) to the IST protocols. It is licensed in the therapy of immune thrombocytopenia due to megakaryocyte stimulating properties. It can also lead to clinically significant improvements in blood counts in nearly half of the patients with AA refractory to IST, increasing BM cellularity, CD34+ count, and progenitor cells through a direct effect on marrow cells (55–57). The mechanism of ELT action in AA is improving signaling via TPO receptor (*c-mpl*) expressed on the surface of HSCs and progenitor cells irrespective of interferon-γ inflammatory microenvironment in BM, by bypassing inhibition caused by interferon-γ competitively binding to the same receptor (58). In fact, about 45% of patients experienced a hematological response to ELT alone, having not only higher platelet counts but also significant improvements in hemoglobin levels and ANC. Townsley et al. evaluated the efficacy of hATG and CsA with addition of ELT in 92 patients in open-label pivotal study (55). Data were compared with results from a historical group of patients receiving solely hATG with CsA. Overall response at 3 months was 74% and 80% at 6 months in comparison with results obtained in a historical cohort of patients (66% at 6 months, P <0.001).

Current recommendations on the treatment of SAA in children and adolescents were published by the European Working Group of Myelodysplastic Syndrome (MDS) and Severe Aplastic Anemia (SAA) (EWOG-MDS/SAA) in 2023 and are available online (59). For adult patients, the recommendations of the EBMT are most often used (60). There are also national UK guidelines available (61).

## 5 Nontransplant therapeutic options in AA

### 5.1 Evaluation of pediatric recommendations

Thanks to IST combined with hATG and cyclosporine A (CsA), the 5-year OS of patients under 16 years of age increased from 50% before 1990 to approximately 80% today (62–64). In a Polish study, the 10-year OS rate reached 78% in children treated mainly with hATG (65). According to a German study, children with vSAA had a higher rate of complete response (CR) than children with SAA (68% *vs* 45%; P = 0.009). They also showed better survival (93% *vs* 81%; P < 0.001) (66).

Different ATG preparations were used in IST with various biological effects. Lymphoglobulin^®^ (hATG, Genzyme) had been widely used for IST in Europe, but it was withdrawn from the market in 2007. In the absence of licensed hATG, rabbit ATG (rATG) sera were used. However, the effectiveness of rATG treatment was worse compared with hATG. In a randomized study by Scheinberg et al., the 3-year OS after rATG was inferior to that of hATG (76% *vs* 96%) (67). Similarly, the EBMT Severe Aplastic Anemia Working Party (EBMT-SAA-WP) reported that the 2-year OS after r-ATG was only 68% *vs* 86% for hATG (68). Some studies (mainly retrospective ones) showed similar effectiveness of hATG and rATG (68–74). Chang et al. reported the 4-year OS of 75% without statistical difference between patients treated with hATG (n = 29) and rATG (n = 33) (75). In contrast, the EWOG-SAA-2010 interim analysis showed a lower overall response rate (ORR) to IST in patients treated with rATG compared with hATG (22% *vs* 42%, P = 0.03) but without differences in OS (88% *vs* 93%) (76). Similarly, Pawelec et al. demonstrated the 3-year OS of 78% and the 10-year survival of 67% for hATG and rATG, respectively (77). However, in a meta-analysis by Hayakawa et al., the ORR was significantly higher for hATG *vs* rATG (risk ratio [RR], 1.27; 95% CI, 1.05-1.54; P = 0.015) (78). In another meta-analysis by Yang et al., the beneficial effect of hATG on the ORR at 6 months in treatment-naïve patients with AA was also shown (RR, 1.86) with comparable safety profiles (79). A summarized comparison of studies with hATG and rATG in IST of AA patients is shown in **Table 1**.

**Table 1:** Studies comparing hATG and rATG for the treatment of aplastic anemia.

Reference	Design	AA severity	Age range (median) years*	Other agent	hATG (n)	rATG (n) †	Definition of response	hATG response	rATG response	P value
PROSPECTIVE STUDIES										
Zheng Y. et al., Exp Hemat. 2006	Prospective, randomized	SAA/VSAA‡	2-71 (34)	All: androgen (stanozolol or testosteron) Regimens I hATG II hATG+CsA III hATG+CsA+rhuGM-CSF+rhuEPO IV rATG+CsA+rhuGM-CSF+rhuEPO	47 (LG)	32	6-months ORR (CR/PR)**	79%	53%	p = 0.039
Scheinberg P. et al., N Eng J Med. 2011 (67)	Prospective, randomized	SAA/VSAA	2-77 (28)	CsA	60 (ATGAM®)	60	Hematologic response at 6 months (no meeting SAA criteria)	68%	37%	< 0.001
Marsh J.C. et al., Blood. 2012 (68)	Prospective for rATG	VSAA/SAA/NSAA	17-75 (36)	CsA	105 (LG) matched registry data	35	Best total response (CR/PR)	67%	60%	NS
RETROSPECTIVE STUDIES										
Atta E.H. et al., Ann Hemat. 2010	Retrospective	SAA/VSAA	1-66 (20)	CsA, steroids, G-CSF, rhu EPO, androgens	42 (LG)	29	6-months ORR (CR/PR)	59.5%	34.5%	0.05
Chang M.H. et al., Eur J Haemat. 2010 (75)	Retrospective	SAA/VSAA	15-78 (49)	CsA	29 (ATGAM®)	33	6-months ORR (CR/PR); but for CRs ANC >2 G/L	52%	48%	0.304
Afable M.G. et al., Haematologica. 2011 (69)	Retrospective	SAA/VSAA	3-80 (51)	CsA	67 (ATGAM®)	20	Hematologic response at 6 months (no meeting SAA criteria)	58%	45%	0.44
Shin S.H. et al., Ann Hemat. 2013 (70)	Retrospective	SAA/VSAA	14-75 (36)	CsA	46 (LG)	53	6-months ORR (CR/PR)	39.1%	45.3%	0.537
Yoshimi A. et al., Blood. 2013 (76)	Retrospective	SAA/VSAA	<18 (9.7)	CsA (+/- G-CSF)	96 (LG)	32	6-months ORR (CR/PR)	65%	34%	0.003
Jeong D.C. et al., Haematologica. 2014 (71)	Retrospective	SAA/VSAA	0-17 (8)	CsA	297 (LG)	158	6-months ORR (CR/PR)	60%	55%	NS
Vallejo C. et al., Ann Hemat. 2015 (72)	Retrospective	VSAA/SAA/NSAA	2-84 (44)	CsA	46 (LG) + 16 (ATGAM®)	169	6-months cumulative incidence of hematologic response	75.4%	73.2%	0.665
Suzuki T. et al., Int J Hemat. 2016 (73)	Retrospective	VSAA/SAA/NSAA	15-75 (51)	CsA	25 (LG)	22	6-months ORR (CR/PR)	56.0%	64.6%	0.39
Vaht K. et al., Eur J Haemat. 2018 (74)	Retrospective	VSAA/SAA/NSAA	2-85 (53)	CsA or tacrolimus	27 (ATGAM® or LG)	128	Best cumulative overall response	51.8%	45.3%	0.536
Cle D.V. et al., Ann Hemat. 2018	Retrospective	VSAA/SAA/NSAA	1-63 (14) hATG	CsA (+/- G-CSF)	85 (LG)	170	6-months ORR (CR/PR)	59%	31%	< 0.001
		VSAA/SAA/NSAA	1-72 (16) rATG	CsA (+/- G-CSF)						
Alashkar F. et al., Eur J Haemat. 2019	Retrospective	VSAA/SAA/NSAA	18-89 (45)	CsA	49 (ATGAM® or LG)	18	6-months ORR (CR/PR)	75.5%	44.4%	0.02
Khan M. et al., J Ayub Med Coll Abbottabad. 2022	Retrospective	VSAA/SAA/NSAA	2-97 (23)	CsA	22	68	6-months ORR (CR/PR)	43.7% (SAA)	35.1% (SAA)	NR
		VSAA/SAA/NSAA		CsA			6-months ORR (CR/PR)	33.3% (vSAA)	22.5% (vSAA)	NR
META-ANALYSES										
Hayakawa J. et al., Int J Hemat. 2017 (78)	Meta-analysis	AA	–	All but 2 studies with CsA	728	624	6-months ORR (CR/PR)	RR 1.27 (95%, 1.05–1.54)	for LG RR 1.21 (95%, 0.97–1.52); for ATGAM® RR 1.42 (95%, 1.0–2.0)	0.014
Yang N. et al., Ann Hemat. 2017 (79)	Meta-analysis	AA	–	CsA	921	715	6-months ORR (CR/PR)	OR 3.73 (95% CI, 1.75–7.94)		0.0006

Table adapted from Scheinberg P. Br J Haematol. 2021;194(6):954–69 with update and modification.

Only manuscripts that include >10 patients per group in a comparative analysis between horse and rabbit anti thymocyte globulin (ATG) are shown. Abstracts were not included.

* Median age for the whole cohort (both hATG and rATG) or independently for hATG and rATG, except for Vallejo et al., which depicts where the mean age is given.

** Criteria used in most studies: CRs: transfusion independence, Hb ≥11 or 10 g/dL, ANC ≥1.5 or 1 G/L, PLT >100 G/L; PRs: transfusion independence, Hb >8 g/dL, ANC >0.5 G/L, PLT >20 G/L.

† rATG formulation is Thymoglobulin in all studies except for in Zheng et al., where Fresenius was used, and Khan et al., where it was not specified.

‡ patients with a PNH clone excluded.

CR, complete response; G-CSF, granulocyte colony stimulating factor; hATG, horse anti-thymocyte globulin; LG, lymphoglobulin; NR, not reported; NS, non-significant; NSAA, Non-severe aplastic anemia; OR, odds ratio of a higher response with hATG; ORR,overall response rate; PR, partial response; rhuEPO, rhu erythropoietin; rhuGM-CSF, recombinant human granulocyte-macrophage colony-stimulating factor; RR, the relative risk of a higher response with hATG; rATG, rabbit anti-thymocyte globulin; SAA, severe aplastic anemia; vSAA, very severe aplastic anemia. Red text means statistically significant differences between hATG and rATG use in immunosuppressive protocols.

A third source of ATG – porcine serum (pATG) – was studied in a few Chinese trials. In a study by Bing et al., the ORR was 83.3% (CR, 54.2%; partial response [PR], 29.2%) with a median time of 90 days (range, 23-380), and 10.4% of patients died of infection within 30 days (80). A pediatric study by Zhu et al. revealed a similar therapeutic effect and safety of pATG or rATG combined with CsA (24-month ORR, 81.6% *vs* 78.6%) as first-line therapy for pediatric patients with AA (81). In retrospective studies by Liu et al. and Chen et al., no significant differences in OS between pATG and rATG were observed (82, 83).

Factors increasing the ORR to IST in children include severity (ORR better in vSAA than in SAA), younger age, higher pretreatment reticulocyte count and lymphocyte count, male sex, and leukocyte count (84, 85). In a meta-analysis by Gu et al., the earlier start of IST, higher neutrophil count at IST, and rate of lymphocyte clearance during IST were associated with improved response rates (86).

The addition of granulocyte-colony-stimulating factor (G-CSF) may reduce the rate of early infectious episodes and days of hospitalization in vSAA patients, but has no effect on the response rate to IST and survival (87, 88). The safety of G-CSF after IST might be compromised. Japanese studies from 1998 and 2002 suggested a close relationship between the long-term use of G-CSF and secondary MDS in nonresponders to IST (89, 90). In a study on 802 SAA patients, a multivariate analysis revealed increasing age, higher disease severity, and increasing number of G-CSF therapy days to be risk factors for MDS/AML transformation. However, the prolonged G-CSF administration in these studies was typical for patients with suboptimal response, and this factor can be associated with other, nonimmune mediated mechanisms of SAA pathogenesis and, in turn, with a higher risk of clonal disease (91). A meta-analysis by Ding et al. and a follow-up study of a randomized, prospective trial by Tichelli et al. did not confirm any impact of G-CSF on the risk of clonal evolution in patients with SAA (92, 93). Regardless of the use of G-CSF, SAA patients treated with IST are at risk of several late malignant and nonmalignant complications. There is a significant risk of relapse (10% at 10 years) and 8%–16% risk of clonal disease (MDS/AML), which does not reach plateau (64, 85, 93).

**Figure 1:**
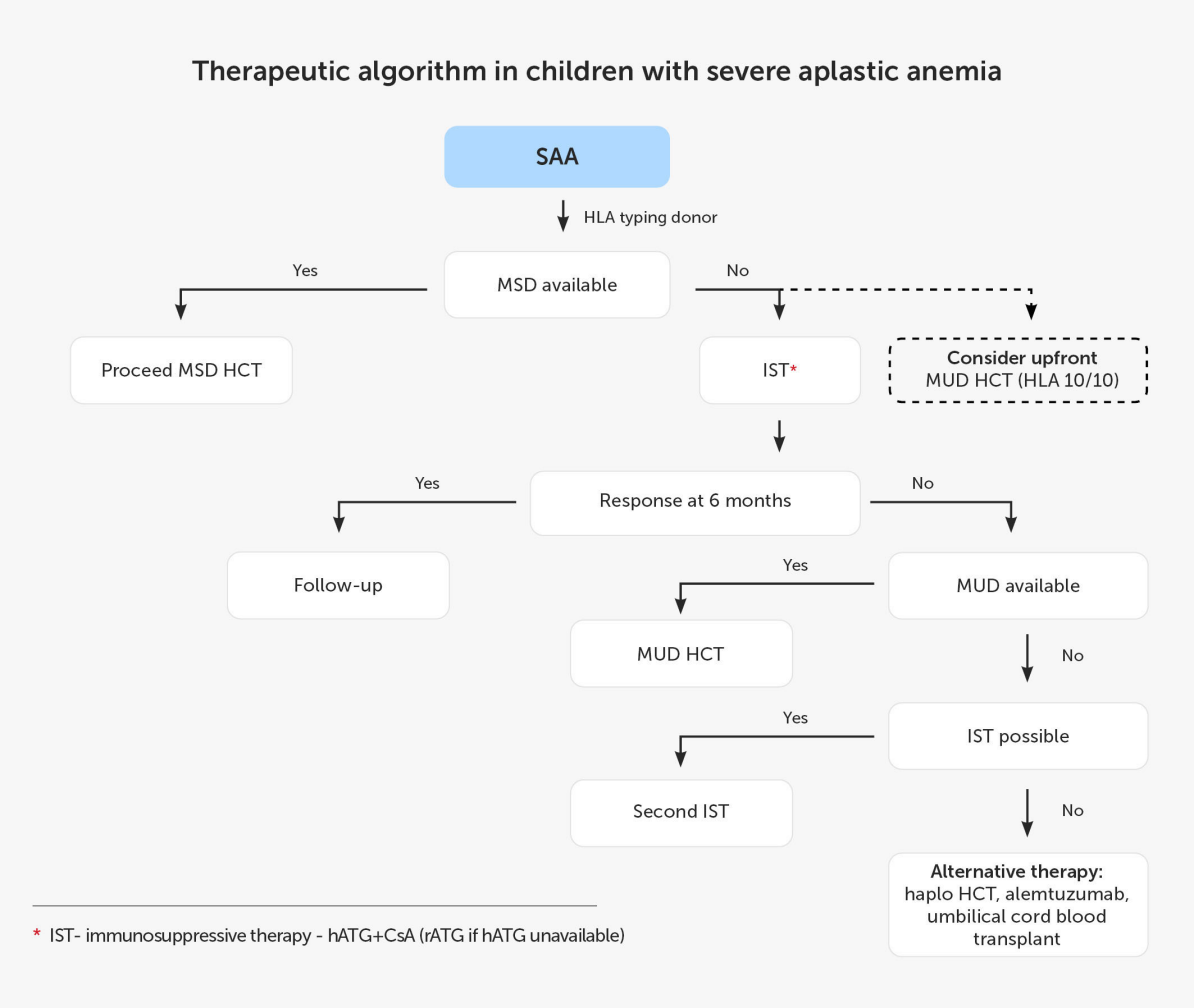
Therapeutic algorithm in children with severe aplastic anemia. CsA, cyclosporin A; hATG, horse anti-thymocyte globulin; HCT, hematopoietic cell transplantation; Haplo, haploidentical donor; HLA, human leukocyte antigen; IST, immunosuppressive treatment; MSD, 8/8 matched sibling donor; MUD, 10/10 matched unrelated donor; rATG, rabbit anti-thymocyte globulin; SAA, severe aplastic anemia.

In the pediatric EWOG strategy for SAA treatment if hATG is not available, rATG may be used as an alternative therapy. Currently, G-CSF should only be used in patients with severe neutropenia (ANC <0.5 × 10^9^/L) and prolonged administration of G-CSF should be avoided. Children not responding to the IST therapy or relapsing afterwards must seek alloHCT from MUDs or alternative donors (ADs) (59). According to the Pediatric Haemato-Oncology Italian Association, patients without MUD can receive the second course of IST (94). A switch of serotherapy from hATG to rATG and vice-versa should be considered, but the expected ORR is only 30% after failure of the first IST and 60% after relapse (95). The remaining options include transplantation from ADs and immunotherapy with alemtuzumab and ELT. The overview of the SAA management in the pediatric population based on the current recommendations is presented in **Figure 1**.

### 5.2 Evaluation of adult recommendations

**Figure 2:**
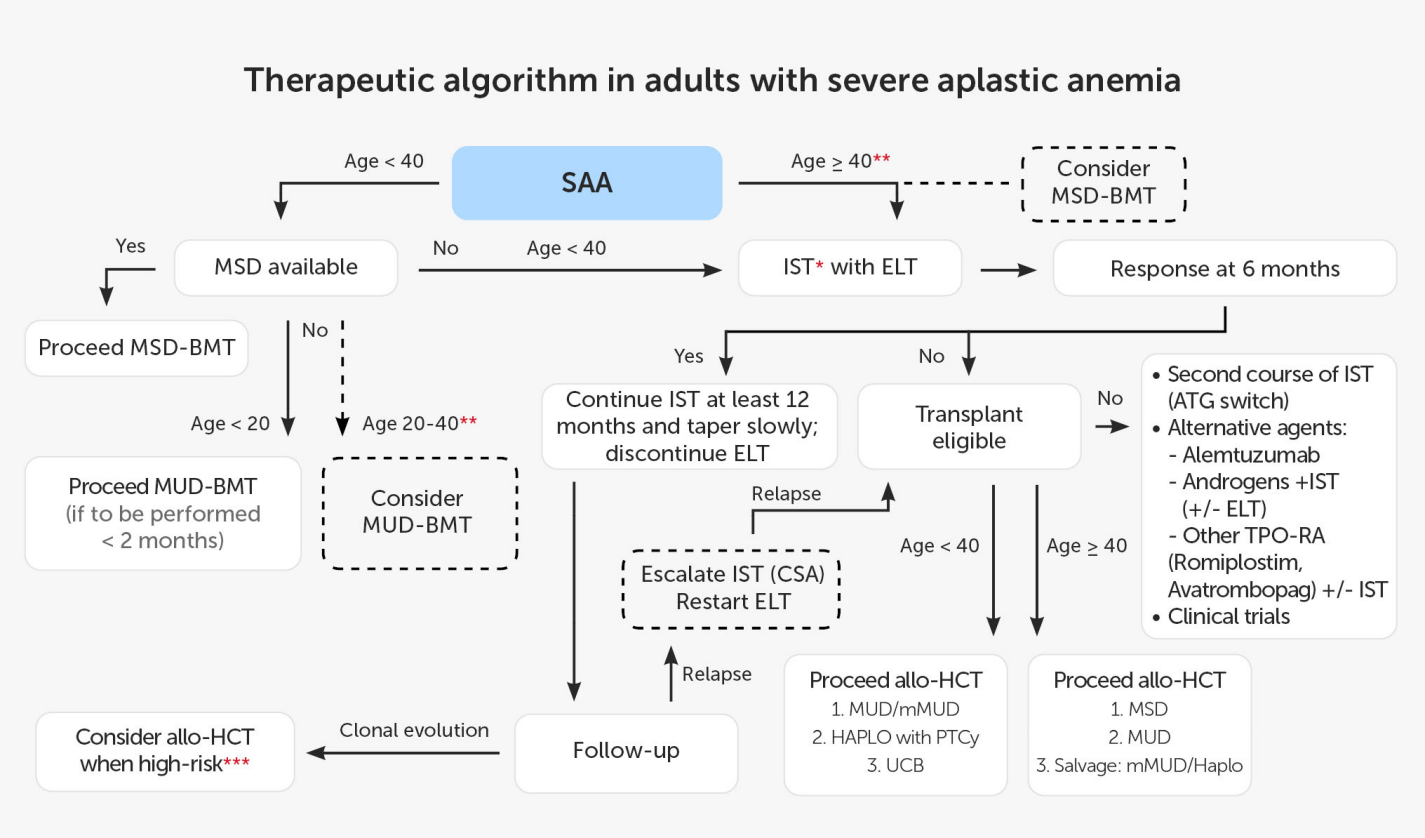
Therapeutic algorithm in adults with severe aplastic anemia. * IST: hATG + CsA (rATG if hATG not available; without ELT if unavailable). ** present predictors of poor response to IST (e.g. age, low reticulocyte count, low lymphocyte count, vSAA, no PNH clone). *** e.g. del7, complex karyotype, dysplasia, increased percentage of blasts). allo-HCT, allogeneic hematopoietic cell transplantation; ATG, anti-thymocyte globulin; BMT, bone marrow transplantation; CsA, cyclosporin A; ELT,eltrombopag; HAPLO, haploidentical donor; hATG, horse anti-thymocyte globulin; IST, immunosuppressive treatment; MSD, 8/8 matched sibling donor; MUD, 10/10 matched unrelated donor; mMUD, 9/10 mismatched unrelated donor; PNH, paroxysmal nocturnal hemoglobinuria; PTCy, post-transplant cyclophosphamide; rATG, rabbit anti-thymocyte globulin; SAA, severe aplastic anemia; TPO-RA, thrombopoietin receptor agonist; UCB, umbilical cord blood;vSAA, very SAA.

According to EBMT recommendations, patients younger than 40 years should undergo alloHCT from MSD. Patients above this age limit and patients without MSD are candidates for IST (60). Best outcomes are reported with hATG combined with CsA and ELT, and this triple combination is currently recommended in adult patients. The current treatment schedule for patients with SAA based on EBMT recommendations and data from the available literature is shown in **Figure 2**.

A standard total dose of hATG (ATGAM^®^) is 160 mg/kg. Most studies evaluated the dose given in a 4-day schedule (40 mg/kg daily for 4 days). The dose was evaluated in 2 prospective randomized studies by Scheinberg et al.: one study evaluating the use of hATG plus CsA with or without sirolimus and the other comparing hATG with rATG (67, 96). Cyclosporine A was given orally at an initial dose of 5 mg/kg for several months (34, 61). In earlier studies, ATGAM^®^ was given at lower daily doses for 8 or 10 days, but at the same total dose of 160 mg/kg. Champlin et al. evaluated hATG given for 8 days (20 mg/kg daily) in monotherapy or with androgens (50). Doney et al. used hATG for 10 days in combination with oxymetholone given orally for 3 months (97). In both studies, CsA was not incorporated in IST protocols, so it is impossible to compare the outcomes with those obtained in modern studies. A lower total dose of hATG was evaluated in a retrospective trial by Scott et al. Two schedules of IST based on CsA and hATG were compared: one group (n = 14) received hATG at a standard dose of 40 mg/kg daily for 4 days, and the other group (n = 17) received 15 mg/kg daily for 5 days (98). The efficacy of both treatment regimens was comparable, although a low number of patients and the retrospective trial design should be considered.

Best outcomes were obtained when hATG was combined with CsA, resulting in 60%-65% of responses, including 10% of CR (96, 99). The addition of other immunosuppressive agents, including sirolimus and mycophenolate mofetil (MMF), did not improve the response rate in prospective randomized trials (52, 96, 100). A retrospective multicenter real-life study included all patients diagnosed with AA between 2000 and 2011 in Sweden and treated in the first line with ATG plus CsA. The study evaluated 158 patients, 128 of whom received rATG. Response to treatment was reported in 47% of patients. The 5-year OS was significantly higher in patients achieving PR or CR as compared with nonresponders (89% *vs* 52%, P <0.001). The 5-year OS was 77.3% and 58.5% for SAA and vSAA, respectively (P = 0.002). The age of patients did not influence the efficacy of treatment (74). The EBMT performed a retrospective study evaluating the efficacy of rATG in Europe and Asia. The study included 955 patients receiving the first-line treatment with ATG and CsA between 2001 and 2012. The ORR was 37% at 3 months and 52% at 6 months, with a subsequent increase up to 65% at 12 months after treatment. The OS at 10 years was 70% (101).

The prospective randomized trial by Scheinberg et al. revealed superior efficacy of hATG *vs* rATG (67). A total of 120 patients were evaluated, 60 patients per group. The ORR at 3 months of IST was 62% in the hATG group and 33% in the rATG group (P = 0.0017). The response evaluated at 6 months was also superior for hATG compared with rATG (68% *vs* 37%; P <0.001). The 3-year OS was also significantly higher for hATG than for rATG (96% *vs* 76%, P = 0.04). Real-world data from France concerning the efficacy of hATG in SAA were also encouraging (102).

The safety issues concerning the infusion of either hATG or rATG are similar, as the most common complications include skin reactions, headache, chills, and fever. In rare cases, anaphylactic reactions or systemic inflammatory reactions due to cytokine release may occur such as cytokine release syndrome or systemic inflammatory response syndrome. To reduce the incidence of immediate adverse reactions, corticosteroids, antipyretics, and antihistamines are recommended (60). To reduce the risk of infusion-related side effects, the preinfusion skin testing should be considered, especially in patients with an increased risk of anaphylaxis (e.g. patients with atopic eczema). However, the negative results of the skin testing do not exclude anaphylactic reactions or development of serum sickness. To reduce the risk of thrombophlebitis, ATG administration via central line is recommended. The prolonged infusion time (minimum 12 hours) is linked to better infusion tolerance (60). Several days after ATG infusion, serum sickness may occur including rash, arthralgia, and fever. The symptoms respond well to steroid treatment. A temporary increase in liver function tests may be observed after ATG administration. The most frequent severe adverse events were infections and hemorrhage (67, 96, 102).

Best response to IST should be evaluated 4 to 6 months after hATG administration (1, 60, 61, 103). The criteria of CR used in recent clinical trials are as follows: ANC >1 × 10^9^/L, hemoglobin >10 g/dL, and platelet count >100 × 10^9^/L (96). Most patients achieve PR when they no longer meet criteria for severe type of AA and become transfusion independent but do not meet CR criteria (ANC >0.5 × 10^9^/L, Hb >8 g/dL, platelet count >20 × 10^9^/L). Long-term responses to IST are observed in 60% of patients. However, some patients require more prolonged CsA treatment exceeding the minimal recommended 12 months and continuation of CsA for a longer time with subsequent slow tapering to minimize the risk of relapse (34). In 10% to 30% of patients, SAA may recur after initial response (1, 60, 61, 103). In such cases, alloHCT should be performed if possible or an alternative strategy with repeating IST might be considered, depending on the individual patient risk assessment and donor access. The second IST pathway should incorporate the option of the change of ATG origin or the use of alemtuzumab and addition of TPO-RA; however, insufficient stem cell reserve might limit the efficacy of IST (34, 52, 60, 61, 95, 104).

### 5.3 Thrombopoietin-mimetic therapy and immunosuppression

In adult patients, ELT proved to be an effective agent in patients with AA refractory to previous IST. Desmond et al. evaluated 43 patients unresponsive to IST and observed long-lasting responses in 40% of cases (56). In the prospective randomized RACE trial conducted by the EBMT-SAA-WP, 101 patients in the hATG + CsA arm and 96 patients in the hATG + CsA + ELT arm were evaluated (33). The median patient age was 53 years, and only 9 patients were children. The dose of ELT was 150 mg daily from day 14. The results were in favor of ELT addition in the first-line treatment with a significantly higher CR in the group receiving ELT at 3 months (22% *vs* 10%; P = 0.01) and a higher ORR at 3 months (59% *vs* 31%) and at 6 months (68% *vs* 41%). The ELT group responded faster to treatment and had no additional toxicity, including hepatotoxicity. The 2-year OS was comparable in both arms. The incidence of somatic mutations was similar in both groups and increased at 6 months after the administration of hATG, with clonal evolution observed in 10% to 15% of cases (33). In a Chinese study, the beneficial effect of prolonged ELT administration was demonstrated in patients without CR at 6 months (105). Based on data from these pivotal studies, the current standard approach to the therapy of SAA in adults includes the combination of IST with hATG with addition of ELT.

However, the effects of ELT on pediatric patients with SAA are less well documented and remain controversial, and the evidence is conflicting. Among the pivotal study population, 21% of patients were children aged 3-18 years, but in contrast to adults, in the pediatric sub-cohort analysis by Groarke et al., in children treated with ELT plus IST there was no significant difference in either the ORR or CR rate at 6 months (ORR: 70% in the ELT group and 72% in the historical group; P = 0.78) (57). In a study by Fang et al., better responses at 6 months were seen in children treated with ELT plus IST (CR: 50% *vs* 17.9%, P < 0.05; ORR: 94.4% *vs* 69.2%; P < 0.05) (106). Goronkova et al. reported a similar ORR in the ELT + IST and IST groups (65% *vs* 53%; P = 0.218). However, the CR rate was significantly higher in the ELT + IST group (31% *vs* 12%; P = 0.027) (107). Of note, the greatest benefit from ELT combined with IST was observed in patients with SAA with ORR 89% *vs* 57% (P = .028) but not in those with vSAA (52% *vs* 50%; P = .902). Other studies conducted in a small number of patients confirmed more rapid hematological responses and CR rates but failed to show ORR and survival advantage in the pediatric population (108). As there are no sufficient data to support the advantage of combined treatment of IST with ELT, the EWOG does not generally recommend this therapy in children (59).

Apart from ELT, also other TPO-RA are investigated in the SAA setting (109). Romiplostim, an Fc-peptide fusion protein administered subcutaneously, activates intracellular transcriptional pathways via the TPO receptor (110). In the dose-finding and long-term treatment phase 2 trial in SAA patients refractory to IST, 10 patients (30%) maintained a platelet response at 2 and 3 years, including 9 and 5 patients with erythroid and neutrophil response, respectively, without significant toxicity. A romiplostim dose of 10 μg/kg once weekly was suggested as a recommended starting dose (111). In a phase 2/3 study in 31 patients with SAA refractory to IST, a romiplostim dose was titrated by platelet response from 10 μg/kg once weekly up to a maximum of 20 μg/kg, and any hematological response at week 27 and 53 was 84% and 81%, respectively (112). In a retrospective study of 10 patients switched to romiplostim (20 μg/kg/week) after failure of the maximum ELT dose, 7 patients achieved neutrophil, erythroid, or platelet response, including one CR at 12 months (113). There is an ongoing phase 2 clinical trial in patients with SAA (either treatment-naïve or relapsed/refractory) with avatrombopag, a second-generation oral TPO-RA, administered from day 1 until day 180 at a dose of 60 mg/day, with dose adjustment according to platelet count, in combination with IST (hATG and CsA) or without IST (114).

### 5.4 Other nontransplant options in AA

Androgens have been used in AA for decades. Their mechanism of action in BMF is complex. Androgens induce erythropoiesis by increasing erythropoietin secretion and response to erythropoietin; they also stimulate the expansion of hematopoietic progenitors (50). Androgens also act by ameliorating telomerase activity in HSCs, resulting the in prevention of telomere attrition both in acquired SAA and in constitutional BMF syndromes (15, 115, 116). Some androgens have additional immunosuppressive mechanisms. For example, danazol inhibits interleukin-1 and TNF-α secretion by human monocytes in a dose-dependent manner (117). Several studies, including one randomized trial, showed the benefit of adding androgens to IST (34, 51, 118). Combined therapy with an immunosuppressive agent, ELT, and androgens in refractory SAA was also reported, with an ORR of 42% (119). The recommended doses for androgens in SAA are 2.5 mg/kg/d for oxymetholone and methenolone and 1 mg/kg/d for methandrostenolone and norethandrolone (46, 120).

Alemtuzumab (a humanized monoclonal antibody directed against CD52 protein) is one of the lymphocytotoxic serotherapeutic options for the treatment of autoimmune diseases. However, in SAA the best results for alemtuzumab were obtained in the relapse and refractory settings, with an ORR of 56% and a 3-year OS of 86% (121).

## 6 Hematopoietic cell transplantation

### 6.1 Challenges in pediatric HCT

AlloHCT in AA is associated with several challenges that have been overcome during the last 40 years. The major concern in AA was the high risk of graft rejection, which was related to the presence of stem cell–eliminating T-lymphocytes and high sensitization towards HLA antigens in heavily transfused patients. The benefit of nonmyeloablative but intensive immunosuppressive transplant protocols with hATG and cyclophosphamide (Cy) was proved in transplantations from MSD in the 1990s (122, 123). Similarly, Cy with rATG-based serotherapy showed very good outcomes with high engraftment rates and mortality associated mostly with invasive fungal infections (124). Improved survival after alloHCT from MUDs was associated with improvements in donor typing, less toxic conditioning regimens with omission of total body irradiation (TBI), and use of leukodepleted blood products (125–127). Advances in supportive care with the availability of new antifungals and effective management of viral infections were pivotal in the reduction of transplant-related mortality.

One of the unresolved controversies in the field of pediatric AA is the problem of upfront HCT from MUDs. Some of the previous evidence supports the benefit of an upfront MUD-HCT in SAA, but randomized clinical trials are needed to confirm such an approach. The analysis of studies conducted between 1998 and 2019 that compared the outcomes of alloHCT and IST as an upfront therapy in SAA showed improved OS in upfront transplant recipients (128). In another study, the outcomes of upfront MUD-HCT in pediatric SAA were similar to those observed for MSD-HCT and superior to those for IST and MUD-HCT performed after IST failure (129). Conducting a randomized controlled trial in the setting of SAA poses a significant challenge. The North American Pediatric Aplastic Anemia Consortium and the Pediatric Transplantation and Cellular Therapy Consortium presented the results of a pilot study randomizing patients to upfront MUD-HCT or IST (130). In this study, 23 of the 57 patients with confirmed SAA underwent randomization and received therapy with a median follow-up of 18 months. This can be seen as a promising herald of high-quality evidence for the upfront therapy of SAA.

### 6.2 Challenges in adult HCT

Transplantation options in adult patients with AA include “upfront” or “delayed” alloHCT after failed IST. The outcomes of alloHCT in AA improved in the last decade with long-lasting OS reaching 70% to 90% (131). On the other hand, despite the improved response to the new immunosuppression protocols with hATG and ELT, some patients are not cured or remain at risk of aplasia relapse or clonal evolution (33). About 3 months after successful IST, in about 19% of patients, chromosomal aberrations, including chromosome 7, were detected. There is also a significant risk of progression to MDS or AML within a median time of 4 to 6 years (27, 31, 55, 103, 132). Therefore, according to the available evidence, the decision to proceed to alloHCT should be based on the benefit-risk analysis of IST *vs* alloHCT. Accessible factors increasing the probability of response to IST and the risk of clonal evolution should be considered in decision-making.

Evaluated biomarkers potentially predictive of better response to IST are as follows: baseline absolute lymphocyte count ≥1 × 10^9^/L; absolute reticulocyte count ≥25 × 10^9^/L; *PIGA*, *BCOR*, and *BCORL* mutations. There are also potential biomarker candidates requiring clinical validation: predominance of memory/activated phenotype of T regulatory cells; increased intracellular IFN-γ levels in circulating T cells; IFN-γ +874T/A gene polymorphism; bone marrow enrichment of CD8^+^ T cells and T cell–activating intercellular interactions; and miR-150-5p expression (31, 133–138). Poor response to IST might be expected with an undetectable PNH clone and a shorter telomere length, and detection of adverse genetic mutations: *ASXL1*, *DNMT3A*, *RUNX1*, *TP53*, but also with TPO plasma levels of >1,796.7 pg/ml (32, 139, 140). Telomere shortening and the above adverse mutations have also unfavorable impact on relapse incidence and clonal evolution (14, 140). If germline mutations were detected, the family history should be carefully collected to determine the malignant hematologic disease germline predisposition because for these patients alloHCT is usually needed.

Factors determining the choice of the therapeutic pathway include the patient’s age, comorbidities, availability of an optimal donor, AA severity at diagnosis as well as access to key medical agents effective in nontransplant therapies: hATG and ELT (26). In a study published by the EBMT, the identified risk factors for alloHCT that should be considered were as follows: PB as the source of hematopoietic cells, time interval to alloHCT exceeding 6 months from diagnosis, patient’s age ≥20 years, no ATG use in conditioning regimen, and the cytomegalovirus (CMV) donor/recipient serostatus other than negative. The presence of risk factors determined inferior survival assessed as 77% and 67% in the intermediate and high-risk groups, respectively, compared with 90% in the low-risk group (141). However, the last risk factor is of a lower value due to a broader access to letermovir prophylaxis in CMV seropositive patients. In another study conducted in patients who underwent transplantation with the use of fludarabine (Flu), the risk factors leading to worse OS included age ≥20 years, shorter time interval (3 months), transfusion burden (>20 units of packed red cells, >50 platelet concentrates), and previous alloHCT. However, in a multivariate analysis, all factors but age were associated with poor survival prognosis (142). Therefore, with the use of Flu, age as a sole risk factor should not be treated as an absolute contraindication to alloHCT. In the study by Sheth et al., the outcome of patients transplanted with FCC conditioning protocol (Flu, Cy, and alemtuzumab) was summarized, and no significant differences were found between patients younger and older than 50 years, but alloHCT outcomes were much worse for patients with a HCT–comorbidity index score (HCT-CI) higher than 3 (143).

For adult patients at the age of 40 years or younger without significant comorbidities, alloHCT from MSDs remains the best option. For older patients, due to higher mortality rates, IST is recommended (60, 144, 145). Nevertheless, some recently published papers showed no significant differences in OS between the age groups when conditioning regimens are based on Flu (143, 146). However, it should be noted that the risk of complications related to graft-versus-host disease (GVHD) is much higher in elderly patients; therefore, a decision to perform upfront alloHCT from MSD in selected patients should be individualized (147). The transplant pathway in the older age group may be recommended for patients with a good performance status and a low HCT-CI in the case of SAA or vSAA and a lack of factors increasing the probability of response to IST (26). Considering disease severity, according to the published data, patients with SAA and vSAA more rarely respond to IST compared with those with nonsevere (moderate) AA. Moreover, they more often experience life-threatening infections and bleeding complications (133, 148).

In the last decade, we observe improved outcomes of alloHCT from MUDs, also in patients with AA (149). It may be explained by better high-resolution donor typing, improved supportive care, and the use of Flu and rATG or alemtuzumab in conditioning protocols with the avoidance of higher doses of TBI and PB as a source of hematopoietic cells (26, 34). However, transplant centers without extensive experience with alloHCT from MUDs and ADs should transfer patients to more experienced centers if such a procedure is planned.

For patients younger than 40 years without MSD, IST is the preferred frontline option; however, in young adults (<20 years) with urgent indications to alloHCT (e.g. recurrent infections) and an available MUD within 8 to 12 weeks, serious consideration should be given to this option (26). In retrospective analyses, the results of alloHCT from MSDs and MUDs were comparable in the population of children and teenagers (129). Another factor to determine opting for upfront alloHCT from MUDs would be the detection of a genetically unfavorable clone increasing the risk of progression to MDS or AML. Early alloHCT from MUDs is the option in patients older than 40 years with failed IST, especially in the case of clonal evolution. However, in the older age group, worse outcomes can be expected, including lower OS and disease-free survival resulting from transplant-related mortality, GVHD, and graft failure (GF) (1, 60, 61). The studies on transplantation techniques and alloHCT outcomes for the treatment of SAA are summarized in **Table 2**.

**Table 2:** Key studies on transplantation techniques and alloHCT outcomes for the treatment of aplastic anemia.

Reference	Number of patients	Years of Tx	Age range (median)	Conditioning	Serotherapy	Donor type	aGVHD incidence	cGVHD incidence	Graft material	Outcome	Comment
Bacigalupo A et al., Haematologica. 2015 (141)	1448	2005-2009	children and adults, 53% >20 years	various	various	MSD 64.9%, MUD 35.1%	25%	26%	BM 26%, PB 42%	OS: MSD - 83%, MUD - 76%	EBMT registry; worse outcome for PBPC, time to Tx, no ATG, age>20yr, CMV status
Chaudhry et al. Biol Blood Marrow Transplant. 2019 (142)	147	2002-2018	3-54	Flu-Cy	ATG	MSD 100%	11.6%	12.9%	BM 52%, BM+PB 45%	OS 83.7%, GVHD-free relapse-free survival 70.7%	Results better with Cy 120 to 200 mg/kg than with higher doses
Sheth VS et al., Blood Adv. 2019 (143)	65	2007-2018	17-71	Flu-Cy	alemtzuzumab	MSD 21.5%, MUD 78.5%	5%	14%	BM 10%, PB 89%	older >50 yr. vs younger: I-year GRFS 84% vs 94%, 5-year OS 86% vs 96%, 1-year TRM 14% vs 5.4%, all results p=ns	Overall survival with an HCT-CI of at least 3 was lower compared with a score less than 3
Samarasinghe S, et al., Am J Hematol. 2019 (150)	261	2000-2013	n/a	Flu-Cy (65%), cyclophosphamide (10 %)	alemtzuzumab	MSD 26.1%, MUD 73.9%	6.7% grade III-IV	16.9%	n/a	5-year OS 81%	EBMT registry; alemtuzumab reduced the risk of acute and chronic GvHD compared with ATG
	1283	2000-2013	n/a	Flu-Cy (20%), cyclophosphamide (52%)	ATG	MSD 66.4%, MUD 33.6%	13.3% grade II-IV	22%	n/a	5-year OS 80%	EBMT registry; alemtuzumab reduced the risk of acute and chronic GvHD compared with ATG
Marsh JC et al., Bone Marrow Transplant 2014 (151)	155	1999-2009	1.5-67.5 (20)	Flu-Cy (60%)	alemtzuzumab (64%), ATG (35%)	MSD 56%, MUD 39%	24%	11%	BM 69%, PB 25%, BM+PB 4%	5-year OS: alemtuzumab 90%, ATG 79%	
Kang HJ et al., Biol Blood Marrow Transplant. 2016 (152)	29 (study B)	n/a	6.4-19.8	Flu-Cy	ATG	MD 51%, MMD 48%	3.5% grade III-IV	37.9%, extensive 17.2%	BM 17%, PB 82%	5-year OS 96%	
Salamonowicz-Bodzioch M et al., JCM 2021 (153)	56	2008-2020	0.8-17.8 (9.4)	Flu-Cy	ATG	MSD 30%, MUD 70%	41.5%, 14.3% grade III-IV	14.2%	BM 30%, PB 70%	5-year OS 94.1%, 2-year GFFS 76.1%	
Darrigo LG et al.,Pediatr Transplant. 2019 (154)	106	2010-2014	children, median 10 yrs.	various	ATG (64%)	MRD 65%, MUD 35%	8%	14%	BM 100%	4-year OS 77%	
Yoo JW et al., Transplant Cell Ther. 2022 (155)	134	2006-2020	children	Flu-Cy	ATG	MD 67%, MMD 32%	4-34%	5.3-7.2%	BM 18%, PB 82%	5-year OS 93%, GFFS 77.5%	
Devillier R et al. Haematologica. 2023 (156)	209 (upfront)	2005-2016	1-64 (21)	chemotherapy	ATG (78%)	MRD 100%	n/a	n/a	BM 72%, PB 28%	5-year OS 88%, GFRS 77%	EBMT registry: the use of ATG/alemtuzumab was associated with a reduced risk of aGvHD, and the use of TBI – with a reduced risk of GF
	270 (relapsed and refractory)	2005-2016	1-77 (27)	TBI (60%), chemotherapy	ATG (68%)	MRD 47%, MUD 53%	n/a	n/a	BM 76%, PB 24%	5-year OS 73%, GFRS 61%	
Chorão P et al., Transplant Cell Ther. 2023 (157)	23	2013-2019	3-63 (37)	cyclophosphamide	ATG	haploidentical	0%	9%	T-cell depleted PBPC	5-year OS 96%	CD34 enrichment as ex vivo depletion
Prata PH et al., Bone Marrow Transplant. 2020 (158)	33	2010-2017	2.5-45.4 (20.4)	PTCy, Baltimore (48%), other (51.5%)	no	haploidentical	23%	10%	BM 51%, PB 42%, BM+PB 6%	2-year OS 78%	
DeZern AE et al., Blood. 2023 (159)	27 (upfront)	2016-2020	3-63 (25)	PTCy, Baltimore with 2 Gy (21%), Baltimore with TBI 4Gy (79%)	ATG	haploidentical	7%	4%	BM 100%	3-year OS 92%	
Gong S et al., Transplant Cell Ther. 2023 (160)	71	2016-2021	9-22 (16)	Flu-Cy + Busulfan + PTCy	no	haploidentical (85%)	4.5%	17.2%, extensive 3.1%	PB 100%	3-year OS 93%	

aGVHD, acute graft-versus-host disease; ATG, anti-thymocyte globulin; BM, bone marrow EBMT, CMV, cytomegalovirus; ESBMT, European Blood and Marrow Transplantation; Flu-Cy, Fludarabine/cyclophosphamide; GF, graft failure; GFFS, GVHD and failure-free survival; GRFS, GVHD-free relapse-free survival; GVHD, Graft-versus-host disease; HCT-CI, Hematopoietic Cell Transplantation-specific Comorbidity Index; MD, matched donor; MMD, mismatched donor; MSD, matched sibling donors; MUD, matched unrelated donors; OS, overall survival; PB, peripheral blood, PBPC, peripheral blood progenitor cell; PTCy, post-transplantation cyclophosphamide; TRM, treatment-related mortality.

### 6.3 Pretransplant management

The patient’s workup before alloHCT should include the assessment of the HCT-CI score, collection of data on previous and current infections (including tuberculosis, viral and fungal infections), and laboratory screening for latent infections. For transfusions, blood products must be irradiated and leukodepleted to avoid alloimmunization against HLA antigens. In patients with significant transfusion burden, serum ferritin should be evaluated. To decrease the risk of organ toxicity, infections, and GF, iron chelation therapy should be considered if the ferritin concentration is higher than 1000 ng/ml.

Fertility preservation issues should be discussed with patients when potentially gonadotoxic conditioning regimen is planned (26, 161–165). The severity of conditioning toxicities can be calculated using a cyclophosphamide equivalent dose (CED) (166). The risk of infertility depends on age and summarized exposure to gonadotoxic chemotherapeutics and radiotherapy (162, 163). The risk of irreversible gonadotoxicity is very high in men receiving a CED higher than 4000 mg/m^2^ and in women receiving a CED higher than 6000 mg/m^2^ (163–165, 167),.

### 6.4 Conditioning regimens

The choice of conditioning regimen in pediatric SAA has evolved over the last 40 years. Among other factors, the omission of initially popular irradiation techniques was associated with progress of HCT and reduced complication rates in this population (168, 169).

Another pivotal step forward in the conditioning was related to the addition of Flu to the MUD-HCT (170, 171). The introduction of Flu into the conditioning regimens of adults with SAA in the last 2 decades was associated with improved outcomes (172). The reduction in the intensity of the conditioning protocol is associated with improved survival but graft-versus-host-free/relapse-free survival (GRFS) might not exceed 80% (152, 153). This issue is especially important in the pediatric population due to long life expectancy of posttransplant children, and the risk of GVHD-associated organ complications and metabolic consequences of life-long steroid immunosuppression that can arise (153).

Twenty years ago, Flu-Cy-ATG greatly improved the outcome of MUD-HCT in children with SAA (173, 174). In the case of MUD-HCT, the optimal conditioning protocol was not evident, but the 5-year OS probability did not exceed 80%, which was inferior to the newer results observed in children. In a retrospective study by the Pediatric Hematopoietic Stem Cell Transplant Working Group of the Brazilian Bone Marrow Transplantation Society and the Brazil-Seattle Consortium (Gedeco), the 4-year OS for MRD-HCT was lower than expected and did not differ significantly from that for MUD-HCT (82% *vs*. 69%, respectively; P = 0.08). The authors highlighted the need for standardized pediatric protocols (154).

Despite the existing variability of transplant protocols in SAA, in 2023 most studies agree that the Flu and Cy chemotherapy backbone is the standard of care for pediatric population. The original Cy dose of 200 mg/kg used in SAA for MSD-HCT can be safely decreased to 100 mg/kg (or 3000 mg/m^2^) in combination with Flu, which results in improved survival, as showed by numerous studies (152, 153, 175). However, at the lower end of the Cy dosing range, in children receiving the dose of 60 mg/kg, an increased risk of graft rejection was observed (176).

A retrospective cohort of 56 children with SAA after Flu-Cy-ATG conditioning showed favorable outcomes (153). The overall incidence of acute GVHD (aGVHD) was 41.5%, and that of grade III-IV aGVHD – 14.3%. Chronic GVHD (cGVHD) was diagnosed in 14.2% of children. The probability of the 2-year GVHD-free survival was 76.1%. In the univariate analysis, a higher dose of Cy and previous IST were significant risk factors for worse OS. Episodes of viral replication occurred in 33 of 56 patients (58.9%), but it did not influence OS. The study showed improvement over the previous treosulfan-based protocol for MUD-HCT (177).

In a Korean study, Yoo et al. described a low rate of secondary GF, higher failure-free survival (FFS), and manageable GVHD regardless of HLA compatibility (155). In 134 children with SAA transplanted with HLA-matched BM transplantation (BMT; n = 24), HLA-matched PB stem cell transplantation (PBSCT; n = 66), and HLA-mismatched PBSCT (n = 44), the estimated 5-year OS, FFS, and GVHD-free FFS of the total cohort were 93.0%, 89.5%, and 77.5%, respectively (155). The incidence of grade II-IV aGVHD was significantly higher in PBSCT than in BMT, but the incidence of grade III-IV aGVHD and cGVHD was similar across all the 3 groups.

In 2023, Vissers et al. described a single-center experience with 228 HCT procedures in pediatric BMF (n = 103) and SAA (n = 125) (178). Patients with SAA transplanted after 2017 had a superior 5-year OS and event-free survival (EFS) of 97% and 85%, respectively, compared with 68% and 59%, respectively, in the cohort transplanted before 2017 (P = 0.0011 and P = 0.017). In the case of BMF, OS and EFS were 89% for those transplanted after 2017 compared with 62% and 59%, respectively, for the other cohort (P = nonsignificant). The authors noticed the typical evolution of the conditioning regimen in SAA, with a change from more toxic to an immune-ablative regimen consisting of Flu/Cy without radiation.

A recent analysis of GRFS by the EBMT-SAA-WP included 479 patients with idiopathic SAA. The probabilities of 5-year GFRS in patients transplanted upfront or in relapsed/refractory cohorts were 77% and 61%, respectively (156). Approximately one-third of all patients were below the age of 20 years, and the group consisted mainly of children. Age was the main risk factor of death (hazard ratio [HR], 1.04; 95% CI, 1.02-1.06; P <0.001), aGvHD (HR, 1.03; 95% CI, 1.00-1.07; P = 0.041), and cGvHD (HR, 1.04; 95% CI, 1.01-1.08; P = 0.032) as the causes of inferior GRFS. The use of ATG/alemtuzumab was associated with a reduced risk of aGvHD (yes *vs* no: 4% *vs* 16%; P = 0.006), and the use of TBI – with a reduced risk of GF (yes *vs* no: 4% *vs* 11%; P = 0.039). There were no significant differences in GRFS and in the causes of GRFS failure according to graft source, donor type, or time from diagnosis to alloHCT. These results suggest that although cured in terms of hematopoiesis recovery, many patients can suffer from detrimental health issues due to long-term sequelae.

The use of MSD-HCT after conditioning with low-dose Cy (80 mg/kg), Flu (175 mg/m^2^), and Thymoglobulin (10 mg/kg) was studied in 32 children with BMF below 14 years of age. While the treatment led to primary engraftment in all patients, 2 patients later developed GF or MDS and only 7 patients sustained donor chimerism above 99% (179). The study was heterogenous and contained 9 patients with constitutional BMF (two *ERCC6L2*, two *ANKRD26*, two *TINF2*, one *LZTFL1*, one *RTEL1*, and one *DNAJC21*), for which Flu/Cy–based conditioning is not optimal.

The chemotherapy backbone recommended by the EWOG for alloHCT in children with SAA consists of Flu (30 mg/m^2^/day, × 4 days) and Cy (25 mg/kg/day, × 4 days) (59). Some small studies indicated that other reduced toxicity protocols, such as Flu with melphalan ± thiotepa, might be used in BMF syndromes (180, 181).

In adult patients, the choice of conditioning regimen is based on the patient’s age and comorbidities, a donor type, evaluation of the risk for graft rejection, access to alemtuzumab, and local experience (26). In the latest evaluation of 955 SAA patients transplanted from HLA-MSD, Flu/Cy/ATG, and Cy/ATG conditioning regimens were associated with the highest survival rates, as compared with Cy ± Flu and busulfan/Cy, with the 5-year probabilities of survival equal to 91%, 91%, 80%, and 84%, respectively (P = 0.001). In the case of 409 recipients of 8/8 and 7/8 HLA allele-matched unrelated donor transplantation (n = 409), the 5-year probabilities of survival with Cy/ATG/TBI-2Gy, Flu/Cy/ATG/TBI-2Gy, Flu/Cy/ATG, and Cy/ATG were 77%, 80%, 75%, and 72%, respectively (P = 0.61) (182). In the MSD setting, Cy-based protocols (200 mg/kg) are recommended in patients younger than 30 years, while in older age groups, Flu (120-150 mg/m^2^) plus Cy (120 mg/kg) are preferred, with the addition of serotherapy in both cases (26, 142, 151, 182, 183). However, in young adult patients at risk of cardiotoxicity, a Flu/Cy-based regimens should be considered. In the 10/10 MUD and 9/10 mismatched unrelated donor settings, low-dose TBI of 2 Gy should be added to the Flu/Cy/serotherapy backbone to decrease the risk of graft rejection (26, 61). In the haploidentical (haploHCT) setting, an increase in the TBI dose to 4-6 Gy may be considered to achieve sustained engraftment (184–186).

### 6.5 Transplantation techniques

The choice of transplant material in alloHCT for SAA is under debate, and resource availability can affect the choice of stem cell source. Bone marrow is considered as the stem cell source for the first alloHCT in SAA because of higher survival probability and lower GVHD incidence (141, 187, 188). In SAA patients, the use of PBSC in MSD was associated with a high incidence of cGVHD of up to 43% (189). The retrospective analysis of 1886 patients with SAA by Bacigalupo et al. showed that BM was superior to PBSC in all age groups and was associated with a lower incidence of aGVHD and cGVHD as well as lower mortality rates (190). However, for technical reasons, the popularity of alloHCT from PB has increased in the recent years (129, 191). In a study by Kumar et al., a multivariate analysis showed the highest OS with BM as graft source in high-income countries compared with PBSCs in all countries or BM in not-high-income countries (P <0.001) (192). The authors concluded that PBSCs may be an acceptable alternative in countries with limited resources when treating patients at high risk of GF and infective complications (193). The transplant outcome in MSD and MUD-HCT for SAA is also improved by the shorter interval from diagnosis to transplantation (127, 128, 141, 190, 194, 195).

To reduce the complication rates, there is a search for ways to improve GVHD prophylaxis. The ex-vivo immunomagnetic T-cell depletion of MSD PBPC is challenging and requires a specialized graft-engineering facility, but it is associated with faster neutrophil engraftment, lower aGVHD incidence, and similar OS as the standard BM (157). However, the T-cell depletion techniques in haploHCT for SAA are associated with an increased risk of GF (196). With the increased data on favorable outcomes with posttransplant Cy (PTCy) as GVHD prophylaxis not only in haploHCT, but also in the MUD and mMUD settings, this option should also be considered (197).

In adult patients, BM is a recommended source of hematopoietic cells in the MSD and MUD settings, but PB might be accepted when alemtuzumab is used (141, 143, 187, 190). The cellularity of the BM graft should be higher than in hematologic malignancies, with the minimal quantity established as 3 × 10^6^ CD34/kg (3 × 10^8^ total nucleated cells [TNC]/kg) (61). In haploHCT, there are no clear recommendations concerning the source and an optimal dose of cells, with a suggestion for a higher dose to diminish the risk of GF. There are different protocols including the preferential use of BM or G-CSF-mobilized BM and PB (186, 198). It must be noted that in the EBMT study no difference in GVHD-free survival was reported when PB was used in haploHCT, with superior platelet engraftment and lower GF rates with a medium graft cellularity of 3.5 × 10^8^ TNC/kg for BM and 7.1 × 10^6^ CD34/kg for PB (158). An option to perform syngeneic HCT should be used irrespective of the patient’s age because of the very good outcomes (long-term survival of about 90%), and in this case, PB is the preferable HSC source (199).

### 6.6 Serotherapy in HCT

The conditioning regimen including serotherapy with rATG, hATG, or alemtuzumab in SAA was shown to be associated with a survival advantage (150, 200). The inclusion of serotherapy with rATG is associated with both reduced risk of cGVHD and increased risk of opportunistic infections in alloHCT recipients (201). In some studies, the benefit of ATG in MSD-HCT was unclear. A multicenter prospective trial by the Center for International Blood & Marrow Transplant Research reported a cGVHD incidence of 21% and 32% in patients conditioned with either Cy alone or Cy/ATG, respectively, while the OS was 74% and 80%, respectively (200). Compared with hATG, rATG was associated with a lower risk of grade II to IV aGVHD (HR, 0.39; P <0.001) but not of cGVHD (182). In a study by Atta et al., rATG was protective against aGVHD and cGVHD, but a higher incidence of invasive fungal disease and mixed chimerism was noted in the rATG *vs* hATG cohorts (202). In another study, 21 patients with SAA, including children, underwent BMT from MSDs following a standard conditioning regimen with Cy (200 mg/kg) and hATG (30 mg/kg/day × 3 days) (203). None of the patients rejected the graft, all had sustained engraftment, and cGVHD developed in 24% of patients. The currently recommended cumulative doses of rATG in children undergoing HCT are 40 to 60 mg/kg for Grafalon and 8 to 10 mg/kg for Thymoglobulin, depending on the donor and grafting material (59).

A strategy to reduce the incidence of GVHD in SAA is the replacement of ATG with alemtuzumab (150, 204, 205). The protocol containing Flu (120 mg/m^2^) and Cy (120 mg/m^2^) with alemtuzumab (40-100 mg) was studied in 50 SAA patients transplanted from MSDs (n = 21) or MUDs (n = 29) (206). Acute GVHD was observed only in 13.5% of patients (all grade I-II), and only 2 patients (4%) developed cGVHD, but GF was present in 9.5% of patients after MSD and 14.5% of those after MUD.

In adult patients, the standard total doses of rATG for SAA in MSD and MUD-HCT are 5 to 10 mg/kg (Thymoglobulin) or 60 mg/kg (Grafalon), but in patients at high risk of graft rejection, a higher Thymoglobulin dose of 10-20 mg/kg might be considered (26, 142). A total dose of alemtuzumab ranges from 40 to 100 mg, and usually 0.2 mg/kg/day is administered for 5 consecutive days (151, 206). In patients referred for alloHCT with alemtuzumab with a high PNH clone associated with SAA, a lowered CD52 expression is possible on the PNH clone-derived lymphocytes as CD52 is a glycosylphosphatidylinositol-anchored protein. Despite theoretical concerns there have been no reports of increased graft rejection or graft failure rates in AA patients with PNH clones using alemtuzumab-containing regimens (207).

### 6.7 Haploidentical HCT

Over the last 20 years, developments in haploHCT with ex-vivo depletion techniques or PTCy gained a lot of attention in multiple indications, including SAA. Nevertheless, SAA HCT protocols should acknowledge the fact that Cy induces cardiotoxicity in 7% to 28% of patients with a mortality rate of 11% to 43% at the therapeutic dose of 170 to 180 mg/kg intravenously (IV). Therefore, caution should be exerted when increasing the cumulative (pre-HCT and post-HCT) Cy dose (208). In patients with a significant risk of cardiotoxicity, a Flu/Cy-based conditioning regimen should be considered. There are also data on the reduction of a standard total PTCy dose of 100 mg/kg to 80 mg/kg (209).

Initial reports on haploHCT showed an engraftment rate of 67% and a 2-year OS of 78% in 33 patients (39% children) transplanted between 2011 and 2017 with PTCy (158). The outcomes of related haploidentical donors after reduced-intensity conditioning with PTCy were presented in 37 patients with SAA: 20 relapsed/refractory and 17 treatment-naïve (186). The 2-year OS probability was 94%, and the cumulative incidence of grade II-IV aGVHD at day 100 and cGVHD at 2 years was 11% and 8%, respectively. The GF incidence was 11% including 1 case in a relapsed/refractory group and 3 in a treatment-naïve patients receiving 2 Gy of TBI while no GF was observed in patients who received 4 Gy of TBI. The role of irradiation cannot be generalized because low-dose TBI/total lymphatic irradiation reduces the risk of GF in adult SAA and is deemed safe, but in children the irradiation techniques, even at a low dose, are not recommended due to excellent transplant outcomes in pediatric SAA and historic reports showing the risk of malignancies in the radiation field (59, 127, 156, 210, 211).

Haploidentical HCT with CD45RA (4 patients), TCRαβ-CD19 depletion or PTCy (5 patients) was evaluated in 11 patients with SAA or MDS-refractory cytopenia of childhood (212). The conditioning regimens were heterogenous and consisted of Flu combined with irradiation techniques (9), Cy (9), busulfan, treosulfan, or thiotepa. Despite engraftment, 2 patients died, and 4 of 9 long-term survivors developed cGVHD.

The clinical protocols for alloHCT in SAA in some countries show fundamental differences, especially by including a high number of partially matched donors and more myeloablative conditioning protocols. Therefore, a direct comparison is not feasible. Older studies that did not implement PTCy after haploHCT revealed an elevated incidence of aGVHD and worse OS probability (213–215). In a study by Qin X et al., in an analysis of 115 patients (mostly children), a conditioning regimen without low-dose irradiation or busulfan was associated with an inferior FFS in a multivariate analysis, but 60% of donors were partially matched in HLA (216). In an Indian study, children and young adults (aged 21 years or younger) with SAA underwent haploHCT after Flu-Cy-ATG/TBI with or without thiotepa/melphalan and PTCy (217). At a median follow-up of 48 months, the OS and EFS were 61.6% and 58.1%, respectively. Chinese protocols in pediatric SAA contain a more intensive chemotherapy backbone, such as Flu (200 mg/m^2^), Cy (30-100 mg/kg), and busulfan (6.4 mg/kg) (160). In a study by Gong et al., 61 of the 71 patients with SAA below the age of 20 years were transplanted from haploidentical donors (160). At a median follow-up of 42 months (95% CI, 33-50 months), the OS and EFS were 93.0% (95% CI, 85.3%-97.3%) and 81.7% (95% CI, 71.5-89.3%), respectively. A more intensive myeloablative protocol resulted in complete donor chimerism in all long-term surviving HCT recipients. In addition, the frequencies of grade II–IV aGVHD, overall cGVHD, and moderate-severe cGVHD were 4.5% (95% CI, 1.3-11.6), 17.2% (95% CI, 9.5-27.8), and 3.1% (95% CI, 0.7-9.6), respectively.

In adult patients, retrospective analyses show minor differences between the outcomes of alloHCT from MUDs and MSDs compared with those of haploidentical donors with the use of PTCy as GVHD prophylaxis. It was suggested that haploHCT should be reserved for younger patients without MSD and MUD after an unsuccessful outcome of IST (34, 104, 218). However, the recent study in mixed age cohort showed encouraging outcomes with haploBMT approach in SAA refractory to IST or relapsed after initial response with 1-year OS 81% and the PTCy-based GVHD prophylaxis. The authors concluded that haploBMT with PTCy could now be considered a standard approach for salvage treatment of SAA (219). Moreover, another prospective study by DeZern et al. enrolling 37 SAA patients with median age 25 (range, 3-63 years) to haploBMT as an upfront approach with PTCy showed OS of 92% at 1, 2 and 3 years with minimal GVHD, while in last 20 consecutive patients receiving a TBI dose increased from 2 Gy to 4 Gy, OS of 100% was achieved (159).

Based on increasing available data, the haploBMT-PTCy platform as the salvage or even initial treatment is of special interest in pediatric and adult patients originating from the ethnical minorities but should be also considered as an alternative option to the MUD/mMUD platform when beneficial outcomes over IST are expected and a delayed alloHCT procedure poses a risk of life-threatening complications.

When donor-specific antibodies (DSA) are identified in pretransplant evaluation of the patient, the preferred haploidentical donor is without antigens corresponding to the detected DSA in the recipient. In the case of a lack of a donor choice and DSA above 1000 MFI, there are 2 options to prevent GF: the search for AD or desensitization of the recipient (220).

### 6.8 Cord blood transplantations

The role of cord blood (CB) in HCT for SAA remains to be determined, although the number of CB transplantations has been on the wane in recent years (221). Increased experience with haploHCT and suboptimal historic outcomes of CB transplantation in SAA suggest that this tendency is becoming stronger (222–224). According to the Chinese study, Haplo-BMT (35 patients) or haploidentical CB (56 patients) HCT based on the Beijing Protocol, with Flu at a dose of 45 mg/m^2^/day on days −7 to −4, busulfan at a dose of 3.2 mg/kg/day IV on days −7 to −6, Cy at a dose of 40 mg/kg/day on days −5 to −2, and rATG (Thymoglobulin) at a dose of 2.5 mg/kg/day IV on days −5 to −2, and GVHD prophylaxis with CsA/tacrolimus with MMF and methotrexate (MTX) was feasible in 91 children (225, 226). The Haplo and Haplo-CB groups had similar 3-year OS (92.8% ± 4.9% *vs* 94.3% ± 3.2, P = nonsignificant), with a lower incidence of aGVHD and cGVHD in the Haplo-CB HCT recipients. In a retrospective analysis of 240 children with SAA transplanted at single center, CB recipients had inferior FFS and GRFS compared with Haplo and MUD cohorts (227). Additionally, CB was associated with a higher GF rate and inferior platelet engraftment. In a study from 2021, HCT in 60 pediatric patients with SAA used unmanipulated haploHCT with third-party 1-5/6 HLA antigen–matched umbilical CB after myeloablative conditioning with busulfan, Cy, and Flu (228). The 5-year transplant-related mortality, OS, and FFS were 2.8%, 97.2%, and 96.2%, respectively. Considering that patients with SAA constituted only a subgroup of 109 patients in the study cohort, the detailed analysis was not provided. It is of note, however, that at +30 days after transplantation, the engrafted HSCs were derived from CB in 5 of the 109 patients. In unique cases, a successful autologous CB transplantation was reported in SAA, but there were differences regarding the use of conditioning therapy before the transplantation (229–232).

The use of CB for transplantation in adults is limited by graft cellularity. Longer time to engraftment is another concern. However, the published outcomes of CB transplantation (CBT) are promising, with an OS of 88% and a low rate of aGVHD and cGVHD of about 15% (233). The APCORD protocol (FluCyATG+2GyTBI) also showed very good results in a prospective trial (222). The recommended minimum TNC dose is 4 × 10^7^ TNC/kg. In a retrospective study of the Japanese Society for Transplantation and Cellular Therapy comparing haploHCT (n = 24) with PTCy *vs* CBT (n = 59) in adult SAA patients, no significant differences were noted in OS, the cumulative incidence of grade II-IV aGVHD, and cGVHD. However, patients after PTCy-haploHCT experienced significantly higher rates of engraftment in neutrophils (95.8% *vs* 78%; P <0.001) and platelets (83.3% *vs* 72.9%; P = 0.025). Moreover, a higher 1-year FFS rate was observed in patients younger than 40 years after haploHCT with PTCy compared with the CBT group (92.9% *vs* 63.9%; P = 0.047) (218).

### 6.9 Posttransplant management and sequelae

The advantages of MUD-HCT include a short time to neutrophil engraftment and high survival rates, but they are counterbalanced by the need for donor search and arrangement of graft donation, which is associated with an up to 4-month delay in therapy. The waiting time for an HSC donor can be used as a window for pharmacotherapy involving IST with or without additional drugs (e.g. TPO-RA or androgens). In addition, HCT can result in treatment-related organ toxicities, infections, GF, poor graft function, acute and chronic GVHD, infertility, and secondary neoplasms, supporting the need for a judicious referral for HCT (234, 235).

The factor with the highest influence on the quality of life is GVHD, and GFRS is the best composite endpoint measuring the success of alloHCT. A risk of GF is another key issue after alloHCT in AA (236). Therefore, the suitable and often individualized posttransplant IST has the most important impact on alloHCT outcome.

Posttransplant GVHD prophylaxis in children typically consists of MTX and CsA, since Locatelli et al. proved this combination to be superior to CsA alone in a randomized controlled trial (237). Due to increased popularity and low cost, PTCy as the sole GVHD prophylaxis after PBSCT from MSDs can be considered, as it is associated with low rates of aGVHD and cGVHD (238).

In adult patients, in the MSD and MUD alloHCT platforms with rATG use, calcineurin inhibitors (CNI) with a short course of MTX is recommended, while alemtuzumab administration is followed by sole CNI-based IST. CNI should be continued for 9 to 12 months with CNI concentrations maintained on higher levels than in hematological malignancy to avoid GF (CsA, 200-300 ng/ml; tacrolimus, 10-15 ng/ml) and very careful tapering under the control of donor chimerism and evidence of GF (61). In haploHCT, there are protocols based on PTCy ± rATG with MMF and tacrolimus given after PTCy administration and the Chinese protocol based on rATG with CsA+MMF starting on day+1 and MTX administrations on days +1, +3, +6, and +11 (186, 239, 240). The APCORD protocol for umbilical CBT comprises CsA at a dose of 200-400 ng/ml for 3 months, with gradual tapering up to 1 year after alloHCT (222).

Donor chimerism is monitored at least 1, 3, 6, and 12 months after alloHCT for the assessment of engraftment kinetics and prediction of GF with progressing mixed chimerism that requires intensification of post-HCT IST (26). Transient mixed chimerism is a common finding after rATG and in the FCC protocol with alemtuzumab, stable mixed chimerism in T lymphocytes indicates that immunotolerance has been achieved (241). In the case of poor graft function with complete donor chimerism, ELT administration or an additional boost of hematopoietic cells without additional conditioning should be considered (242, 243).

In case of alloHCT failure in SAA, second transplants are associated with very high success rates, even with the retransplantation from an original donor (244). In 55 children who experienced GF after the first alloHCT, the 5-year OS and FFS were 82.9% and 81.2%, respectively (245).

The long-term survival of children with SAA after HCT is very high, and the OS probability at 5 and 10 years is 96% and 94%, respectively (246). Changes in the conditioning protocols and improved genetic testing over the last 40 years positively affect the survival outcomes, but caution is warranted. Despite excellent survival probability, the freedom from long-term complications in HCT survivors have not been determined (247). According to Buchbinder et al., the cumulative incidence of late effects among survivors of MSD-HCT was less than 3% and did not increase over time, but after MUD-HCT, the incidence of late effects exceeded 3% by 5 years: gonadal dysfunction, 10.5% (95% CI, 7.3-14.3); growth disturbance, 7.2% (95% CI, 4.4-10.7); avascular necrosis, 6.3% (95% CI, 3.6-9.7); hypothyroidism, 5.5% (95% CI, 2.8-9.0); and cataracts, 5.1% (95% CI, 2.9-8.0). However, the majority of these transplantations included irradiation as part of conditioning regimen (248).

Among the consequences of HCT, the incidence of subsequent malignancies should be considered, estimated at 1.1% among the SAA patients (249). Improved differential diagnosis and genetic testing reduced the misdiagnosis of clonal disorders as SAA and, consequently, the occurrence of myeloid malignancies after HCT (84, 250). It can be suggested that HCT results would further improve in the future due to eliminated or reduced exposure to TBI or alkylating agents. However, long-term surveillance is necessary, because the majority of nonleukemic malignancies developed at least 5 years after transplantation.

## 7 Conclusions

Although the body of evidence on the treatment outcomes in patients with AA is mostly based on retrospective studies and nonrandomized trials, the up-to-date recommendations can be summarized as follows:

1. The diagnosis of patients with AA must include testing for constitutional defects associated with BMF syndromes. Potential related transplant donors must always be tested for constitutional defects if such are found in a patient (proband).
2. Pretransplant care should include transfusions of irradiated leukodepleted blood products as well as anti-infective screening and prophylaxis.
3. Upfront alloHCT from MSD is recommended in children and young adults (age <40 years).
4. Upfront IST with hATG is recommended in elderly patients and children and young adults without MSD and should be performed without delay.
5. Addition of ELT to IST in adults should be considered as a standard of care incorporated in the IST protocols.
6. The option of upfront MUD-HCT can be considered in patients lacking MSD if the donor search and transplantation procedures would be completed within 2 months and the patient is eligible for HCT. However, further studies are necessary to confirm this.
7. Bone marrow is preferred as the source of stem cells due to better outcomes in most transplantation settings.
8. Conditioning regimen for alloHCT should be based on Flu, Cy, and rATG in children; in adults on Cy (age <30 years) and on Cy with Flu (age >30 years), while serotherapy options include rATG or alemtuzumab. In the MUD and Haplo setting in adults, a low dose of TBI should be added to reduce the risk of GF.
9. GVHD prophylaxis after alloHCT should consist of CNI and a short course of MTX or CNI alone when alemtuzumab is used in conditioning.
10. HaploHCT and CBT should not be considered a standard therapy, unless as a salvage after IST failure and the unavailability of MSD/MUD. In the haploHCT setting, PTCy as the GVHD prophylaxis is recommended. Based on increasing available data, the haploBMT-PTCy platform as the salvage or initial treatment might be considered as an alternative option to MUD/mMUD, especially in patients originating from the ethnical minorities.
11. Androgens can be considered as a supportive treatment in patients not responding to IST and ineligible for other therapies.
12. Fertility preservation strategies should be discussed and implemented in patients eligible for alloHCT with potentially gonadotoxic protocols.
13. Long-term surveillance in alloHCT survivors is necessary.

## Author contributions

AP: Conceptualization, Investigation, Methodology, Project administration, Supervision, Validation, Visualization, Writing – original draft, Writing – review & editing. KP: Investigation, Validation, Visualization, Writing – original draft. AS: Investigation, Validation, Writing – original draft. MU: Conceptualization, Investigation, Methodology, Supervision, Validation, Writing – original draft, Writing – review & editing.
